# Hematological- and Immunological-Related Biomarkers to Characterize Patients with COVID-19 from Other Viral Respiratory Diseases

**DOI:** 10.3390/jcm11133578

**Published:** 2022-06-21

**Authors:** Rafael Suárez-Del-Villar-Carrero, Diego Martinez-Urbistondo, Amanda Cuevas-Sierra, Iciar Ibañez-Sustacha, Alberto Candela-Fernandez, Andrea Dominguez-Calvo, Omar Ramos-Lopez, Juan Antonio Vargas, Guillermo Reglero, Paula Villares-Fernandez, Jose Alfredo Martinez

**Affiliations:** 1 Department of Internal Medicine, Hospital Universitario HM Sanchinarro, 28050 Madrid, Spain; rafasdvc@gmail.com (R.S.-D.-V.-C.); dmurbistondo@gmail.com (D.M.-U.); andreadominguezcalvo@gmail.com (A.D.-C.); pvillares@hmhospitales.com (P.V.-F.); 2 IMDEA-Food Institute (Madrid Institute for Advances Studies), Campus of International Excellence (CEI) UAM+CSIC, 28049 Madrid, Spain; guillermo.reglero@imdea.org (G.R.); jalfredo.martinez@imdea.org (J.A.M.); 3 School of Medicine, San Pablo CEU University, 28925 Madrid, Spain; ia.ibanez@usp.ceu.es (I.I.-S.); a.candela2@usp.ceu.es (A.C.-F.); 4 Medicine and Psychology School, Autonomous University of Baja California, Tijuana 22390, Mexico; os_mar6@hotmail.com; 5 Department of Internal Medicine, Hospital Universitario Puerta de Hierro, 28222 Madrid, Spain; juanantonio.vargas@salud.madrid.org; 6 Department of Medicine, Autonomous University of Madrid, 28049 Madrid, Spain

**Keywords:** COVID-19, biomarkers, differential diagnosis, prediction model

## Abstract

COVID-19 has overloaded health system worldwide; thus, it demanded a triage method for an efficient and early discrimination of patients with COVID-19. The objective of this research was to perform a model based on commonly requested hematological variables for an early featuring of patients with COVID-19 form other viral pneumonia. This investigation enrolled 951 patients (mean of age 68 and 56% of male) who underwent a PCR test for respiratory viruses between January 2019 and January 2020, and those who underwent a PCR test for detection of SARS-CoV-2 between February 2020 and October 2020. A comparative analysis of the population according to PCR tests and logistic regression model was performed. A total of 10 variables were found for the characterization of COVID-19: age, sex, anemia, immunosuppression, C-reactive protein, chronic obstructive pulmonary disease, cardiorespiratory disease, metastasis, leukocytes and monocytes. The ROC curve revealed a sensitivity and specificity of 75%. A deep analysis showed low levels of leukocytes in COVID-19-positive patients, which could be used as a primary outcome of COVID-19 detection. In conclusion, this investigation found that commonly requested laboratory variables are able to help physicians to distinguish COVID-19 and perform a quick stratification of patients into different prognostic categories.

## 1. Introduction

Viral pneumonia is one of the main causes of hospital admission worldwide [1]. The main agents of this entity are influenza A and B viruses, rhinovirus, parainfluenza, adenovirus, respiratory syncytial virus, metapneumovirus and bocavirus [2]. Recently, with the COVID-19 outbreak, viral respiratory infection has become a major health threat all over the world [3]. Over six million cases of community-acquired pneumonia occur every year and over 20% need hospital assistance [4]. In this context, SARS-CoV-2 infection has dominated the international epidemiological focus from 2020, which has contributed to an important increase of morbidity and mortality especially in elderly population [5]. In addition, pre-existing complications such as hypertension, obesity, type 1 or type 2 diabetes or cardiovascular diseases have shown to be associated with a greater severity and fatality. In this sense, COVID-19 has even been hypothesized not only as pulmonary, but also a vascular disease [6]. In fact, a systemic inflammatory state can provide a good scenario for viruses such as influenza or COVID-19 [7]. The epidemiological evolution of COVID-19 points towards the coexistence between SARS-CoV-2 and the rest of the seasonal viruses [8]. This fact has led to the clinical challenge of distinguishing between SARS-CoV-2 and other viral etiologies [9]. For this reason, conducting studies comparing the clinical, analytical and prognostic characteristics of COVID-19 with those of other respiratory virus infections could be useful for an effective personalized clinical care [10]. In this sense, the delay in obtaining diagnostic results from PCR-based detection tests—the gold standard in COVID-19 diagnosis—can delay decision-making in terms of management, prognosis and therapy, while antigenic detection of SARS-CoV-2 is limited to specific patients and situations. Therefore, the development of strategies based in clinical determinations to provide not only discrimination capacity of patients with viral pneumonia due to COVID-19 in the hospital setting, but also prognosis and featuring clinical complications, might enhance precision therapeutic and management decisions [11,12,13].

Several demographics, clinical, phenotype or even genetic variables have been described to be associated with the progression of COVID-19 and multisystemic manifestations [14]. Immunological response to the virus can also involve hepatic, gastrointestinal, cardiac, renal, neurological and hematological complications [15]. Liver complications caused by COVID-19 can induce high levels of transaminases during the infection, which in some cases could lead to liver dysfunctions [16]. Proinflammatory markers associated with the response to COVID-19 are easily accessible clinical determinations that could contribute to advance the individualized clinical management and monitoring of this disease and to be integrated in the framework of precision medicine [17]. The quickly increasing number of COVID-19 confirmed cases suggest the requirement of a rapid and effective model of triage based on simple variables to perform hierarchical management of the patients. In fact, the delay of COVID-19 detection can have an important impact on inflammatory status and worsen the prognosis of the disease. In this regard, the use of this routine clinical determinations is crucial for a better understanding of the severity of disease progression that could help to characterize and stratify patients with COVID-19 [18]. Thus, the objective of this study was to develop a statistical model based on hematological variables for the early characterization of patients with COVID-19 from other viral respiratory diseases.

## 2. Materials and Methods

### 2.1. Study Design and Settings

A retrospective and multicenter cohort study was performed including subjects hospitalized due to suspected viral infection. This cohort was carried out including adults male and female patients who were consecutively admitted in the emergency service of the HM Hospitales group in the city of Madrid. This cohort was settled including two groups of patients: the first group included patients who underwent a PCR test for respiratory viruses between January 2019 and January 2020, and the second group comprised those who underwent a PCR test for the detection of SARS-CoV-2 between February 2020 and October 2020. The information of a total of 951 subjects was collected until the deadline in October 2020. Data were collected following current hospital protocols and the study was approved by the Institutional Ethics Board and was carried out in accordance with the Declaration of Helsinki [19] (code: 21.03.1800-GHM). Informed consent was obtained from all subjects involved in the study. The primary outcome defined and followed for this study was to find variables related to COVID-19-positive results.

### 2.2. Data Collection

#### 2.2.1. Participants

This cohort enrolled 951 patients of both genders (419 men and 532 women) with a mean of age of 68, who were consecutively admitted in the emergency system of HM Hospitales group in the city of Madrid (Spain) and who presented a viral respiratory disease. The criteria followed for the inclusion in this cohort were the diagnosis of viral respiratory disease by the physician, adulthood and the admission in the emergency system of HM Hospitales Madrid during the period of time from January 2019 to January 2020 and from February 2020 to October 2020. No participant was included after this date. Patients took part of this cohort after the result of a PCR test. Exclusion criteria were an age less than 18 years, the absence of clinical data and not having obtained a sample result for PCR of respiratory viruses or SARS-CoV-2.

#### 2.2.2. Variables

The database was performed with information of patients at the moment of admission in the emergency system. Variables collected were age, sex, PCR test results with the FDT21 system or COVID-19 PCR according to the RT-PCR method (considering COVID-19, rhinovirus, influenza, parechovirus, adenovirus, respiratory syncytial virus, metapneumovirus and bocavirus), day of hospitalization, days hospitalized in ICU, health complications (cardiovascular and cerebrovascular disease, dementia, cardiorespiratory disease, chronic obstructive pulmonary disease, chronic hepatic disease, chronic kidney disease, hemiplegia, leukemia, lymphoma, solid tumor, metastasis, diabetes type 2, connective tissue disease and human immunodeficiency virus), blood biochemical data (hemoglobin, neutrophils, leukocytes, monocytes, anemia according the WHO definition [20], C-reactive protein and urea), Charlson index calculation, vaccination status, onset of symptoms, oxygen saturation and chest radiography result; additionally, the presence of immunosuppression prior to diagnosis—considered as steroid treatment equivalent to Prednisone at more than 30 mg/kg, treatment with immunosuppressants or chemotherapy in the last 3 months—was collected retrospectively [21,22]. The population was categorized in the database according to the PCR test results into 5 groups: (i) patients with positive SARS-CoV-2 PCR (February 2020–October 2020), (ii) patients with respiratory complications but negative SARS-CoV-2 PCR (February 2020–October 2020), (iii) patients with positive pan-viral PCR for influenza (January 2019–January 2020), (iv) patients with pan-viral PCR positive for rhinovirus, parainfluenza, adenovirus, respiratory syncytial virus, metapneumovirus, bocavirus and non-SARS-CoV-2 coronavirus (January 2019–January 2020) and (v) patients with negative pan-viral PCR (January 2019–January 2020). This last group presented respiratory disease that was not related with any viral infection according to the PCR test.

### 2.3. Statistical Analyses

Results were expressed as numbers of cases and percentages for qualitative variables and the mean and standard deviation for quantitative variable. The normality of analyzed variables was screened with the Shapiro–Wilk test. Statistical differences between groups were assessed by Student’s *t*-test or Mann–Whitney U test and by ANOVA or Kruskal–Wallis test, depending on the distribution of data. The chi-square was performed for the evaluation of qualitative variables and the Student’s *t*-test.

A variable selection procedure was performed for obtaining the best combination of variables capable to discriminate COVID-19 from other viral respiratory disease. A multiple linear regression using the Furnival–Wilson leaps-and-bounds algorithm, specifying the best option (Stata module “vselect”) [23] was carried out. All subsets variable selection provides the R2 adjusted, Mallows’s Cp, Akaike’s information criterion, Akaike’s corrected information criterion and Bayesian information criterion for the best regression at each quantity of predictors. The variable combination with the best R^2^ adjusted was selected for the construction of a logistic regression model to evaluate the predictive capacity for discriminating between patients. Variables were categorized by 0 or 1 according to the presence/absence, while continuous variables were categorized according to the mean (68 years old for age, more than 9000 cells/µL for leukocytes, more than 700 cells/µL for monocytes, more than 110 mg/L for C-reactive protein). Odds ratio values were represented in a forest plot. Based on the logistic regression analysis results, the area under the curve (AUC) from the receiver operating characteristic curve (ROC) was calculated as a validation test of the predictive capacity of the model. A similar logistic regression model was fitted using categorized variables (above or below a cutoff point established by the mean of subjects with fatal outcome) for the construction of a COVID-19 mortality prediction. Additionally, multivariate analyses of the values evaluation were performed using logistic regression. Results with a *p* < 0.05 were considered statistically significant. The SPSS statistical program version 27.0 (Armonk, NY, USA) and Stata 12. (StataCorp LLC, College Station, TX, USA) were used for statistical analyses and figure depiction.

## 3. Results

A total of 951 patients were admitted in the emergency service of HM Hospitales Madrid with suspicion of viral respiratory infection during the period of time mentioned above. A total of 419 subjects were men and 532 were women. The population was categorized in five groups according to PCR results: 313 patients with COVID-19, 119 flu-positive patients, 116 pan-viral-positive patients, 237 COVID-19-negative patients and 166 pan-viral-negative patients. Data related to medical history and hematological determinations according to PCR test results groups are reported in Table 1. A higher number of men subjects were found in COVID-19-positive group.

The mean age of the global population was 68 ± 16 years with 55.9% of men. A 26.1% of the global population presented a cardiorespiratory disease and 23.7% presented anemia as more conjoint complications.

Regarding patients with COVID-19, men presented significantly more cases. The most frequent pre-existing complication was type 2 diabetes in patients with COVID-19 (17.6%), followed by cardiorespiratory disease (14%) and solid tumor (13.1%). Additionally, 8.6% of patients with COVID-19 were immunosuppressed and 22.7% presented anemia (Table 1). In addition, patients with COVID-19 presented more cases of dementia compared with the other respiratory virus and cerebrovascular disease and metastatic tumor, although the differences of cases were not significant between groups.

Interestingly, significant differences were found in chronic obstructive pulmonary disease, in which the pan-viral-positive group presented 22.4% of cases. The total of hospitalization days was higher in COVID-19-positive group compared with other viral respiratory diseases and a higher mortality rate (22.7% vs. 12.7%, *p* < 0.01). Patients with COVID-19 showed a trend towards a longer time of symptoms at admission (6.4 vs. 6.1, *p* = 0.095) with a time of admission similar to the complete population (12 vs. 11 days, *p* = 0.85).

Table 2 shows the hemogram determinations between groups. Hemoglobin levels were higher in patients with COVID-19 (13.5 ± 2 g/dL) as were significant lower total white blood cell counts (*p* < 0.001). The leukocytes and neutrophils levels (cell/µL) were significantly lower in COVID-19-positive group (*p* < 0.001). Similar results were found in monocytes levels. C-reactive protein presented a higher value in COVID-19-positive patients and flu-positive patients, but the difference was not significant (*p* = 0.116) (Table 2).

For addressing the main objective of this work, a variable selection algorithm was applied in order to find the most adequate combination of variable for discriminating between patients with COVID-19 from other viral respiratory disease. The variable selection algorithm obtained the best R^2^ using age, sex, immunosuppression, anemia, C-reactive protein (CRP), chronic obstructive respiratory disease (CODP), cardiorespiratory disease, metastasis, leukocytes level and monocytes level as predictive variables to early distinguish patients with COVID-19.

**Figure 1:**
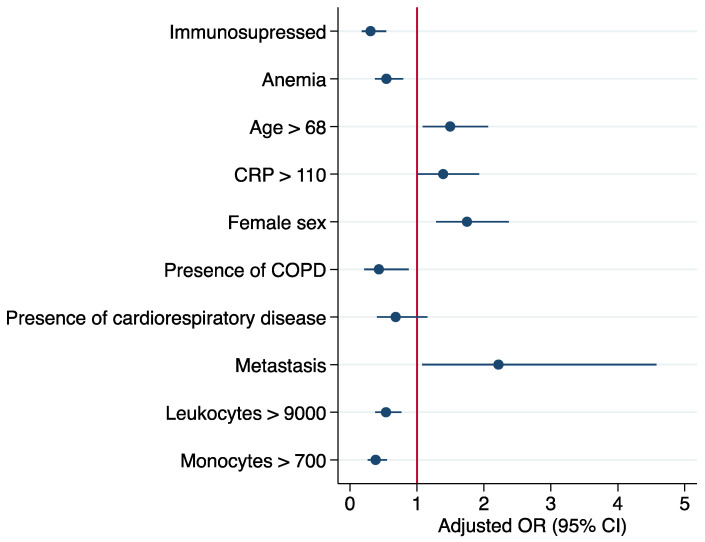
Forest plot of output from the logistic regression model evaluating odds for identifying patients with COVID-19. The circle represents odds ratio value on the *X*-axis. The error bars or whiskers ⊢ ⊣ represent the 95% CI of the odds ratio. The labels on the *Y*-axis represents the variables included in the logistic regression for the identification of patients with COVID-19. Age is expressed as years, C-reactive protein (CRP) as mg/L, leukocytes and monocytes levels as cells/µL. CRP: C-reactive protein; COPD: chronic obstructive pulmonary disease.

**Figure 2:**
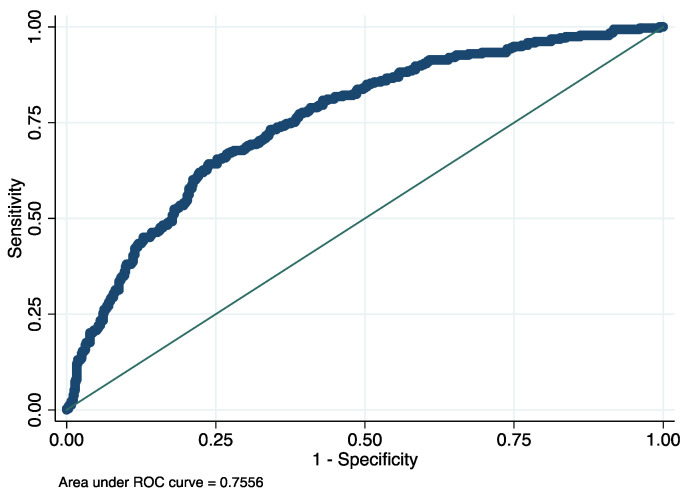
ROC curve of the proposed model showing the discriminant capacity of these variables for distinguishing between patients with COVID-19 from other viral respiratory diseases (AUC = 0.75, *p* value = 0.001).

A logistic regression was fitted using these variables for evaluating the prediction capacity of these inflammatory, hematological clinical outcomes to discriminate patients with COVID-19 (Table 3). Figure 1 show the predictive effect of each variable calculated from the odds ratio. As shown in the graph, the presence of a tumor with metastasis is the main predictive variable for an early discrimination of patients with COVID-19, followed by female sex, high values of CRP and being older than 68. In addition, the absence of immunosuppression, anemia, cardiorespiratory disease and COPD seem to be important variables for the early identification of COVID-19. Low values in monocytes and leukocytes seem to be associated with COVID-19 infection. The depiction of ROC curve analysis performed to estimate the predictive value of the logistic regression proposed, showing a value of 0.75. (AUC = 0.75; *p* = <0.001) (Figure 2).

In line with this results, and since COVID-19 has been previously associated with lymphopenia [24], a complementary analysis of leukocytes’ levels was performed. Patients with COVID-19 resulted in significantly lower values in neutrophils, monocytes and lymphocytes, confirming the presence of lymphopenia in this cohort (Table 4).

**Figure 3:**
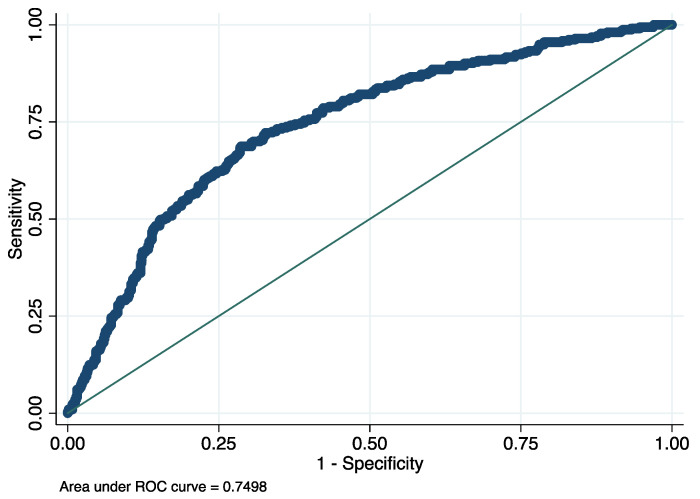
ROC curve for evaluating the discrimination capacity of the logistic model proposed for predicting mortality in patients with COVID-19. AUC = 0.75. *p* value = 0.001.

Taking into account the rates of mortality with COVID-19 infection, a complementary analysis of multivariate model for the prediction of mortality was fitted using clinical, hematological and biochemical variables associated with the severity and mortality of COVID-19, such as age, sex, peripheral oxygen saturation, C-reactive protein, time of onset of symptoms, neutrophils and lymphocytes levels, anemia, cardiorespiratory disease and immunosuppression. These results showed that having low levels of monocytes (<500 cells/µL was the principal variable for detecting high risk of mortality in patients (OR = 2.21; *p* < 0.001) (Table 5, Figure 3).

**Table 5:** Logistic regression model using mortality as the dependent variable and clinical and biochemical determinations with discrimination capacity for exitus in patients with COVID-19.

Variables	Total of Patients *n* = 951	Univariate Analysis OR (CI)	*p* Value	Multivariate Analysis OR (CI)	*p* Value
**Age > 65, %**	577 (60.7)	1.17 (0.89–1.55)	0.253	1.61 (1.16–2.23)	0.004
**Sex male, %**	532 (55.9)	1.56 (1.11–2.06)	0.002	1.73 (1.27–2.36)	<0.001
**Oxygen saturation > 90%, %**	574 (60.4)	1.41 (1.06–1.87)	0.016	1.50 (1.09–2.07)	0.012
**Monocytes < 500 cells/µL, %**	427 (44.9)	2.95 (2.23–3.90)	<0.001	2.21 (1.59–3.08)	<0.001
**Neutrophils < 5500 cells/µL, %**	445 (46.8)	1.72 (1.31–2.27)	<0.001	1.53 (1.09–2.14)	0.013
**Lymphocytes < 1500 cells/µL, %**	723 (76)	2.31 (1.62–3.31)	<0.001	1.69 (1.13–2.52)	0.01
**C-reactive protein > 80 mg/dL, %**	450 (47.3)	1.46 (1.11–1.91)	0.006	1.55 (1.12–2.16)	0.008
**Onset of symptoms > 4 días, %**	473 (49.7)	2.05 (1.56–2.71)	<0.001	1.46 (1.07–1.99)	0.015
**Anemia, %**	315 (33.1)	0.47 (0.34–0.64)	<0.001	0.56 (0.38–0.38)	0.004
**Cardiorespiratory disease, %**	216 (22.7)	0.44 (0.30–0.63)	<0.001	0.47 (0.31–0.71)	<0.001
**Immunosuppression, %**	167 (17.6)	0.33 (0.21–0.52)	<0.001	0.37 (0.22–0.61)	<0.001

## 4. Discussion

COVID-19 disease has affected all international territories causing high pressure in hospitals in all countries and higher fatality rates than other respiratory diseases [25]. The appearance of COVID-19 is usually accompanied by a high inflammation status, which could be used as a prospect to distinct the presence or absence of COVID-19, using also phenotypical, biochemical and medical history variables [26]. Such outcomes suggest that a set of easily available clinical variables might be able to discriminate patients with COVID-19 from those infected by other viruses with a high discrimination power in this population [27]. To our knowledge, this is the first study to show the importance of combining hematological and medical variables for featuring patients with COVID-19.

Regarding the results obtained from the comparison of variables of medical history between groups, we found an average age of 68 years in global population, but 70 in subjects affected by COVID-19. This results are in line with others previous publications that showed that individuals older than 60 years need hospitalization after COVID-19 infection [28]. Pre-existing complications were more common in patients with COVID-19, such as type 2 diabetes or presence of solid tumor, which have been largely describe to be a risk factor for COVID-19 complications in scientific literature [29,30]. Patients with COVID-19 also presented more cases of ischemic heart disease, dementia and cerebrovascular disease compared with the other viral respiratory diseases, highlighting the importance of taking into account the clinical history of the patient to know the evolution of COVID-19. The percentage of exitus was significantly higher in patients with COVID-19 in comparison with the other viral respiratory diseases, showing the importance of diagnostic tools, which can quickly and easily identify the disease before fatal progression.

Regarding the hematological and biochemical determinations, patients with lower respiratory infection caused by COVID-19 had lower levels of white cells (leukocyte, neutrophils, monocytes and lymphocytes cells). Previous studies have found that lymphopenia can be consider an important factor for differentiating between COVID-19 and influenza [31]. The reason why COVID-19 patients presented lower levels of lymphocytes is still unclear, although some hypothesis suggested that COVID-19 increases levels of tumor necrosis factor-α and interleukin-6, which are closely correlated with lymphopenia [32]. In this line, the neutrophils/lymphocytes ratio was included because it is an easily measurable, available, cost-effective and reliable parameter of systemic inflammation, whose continuous monitoring could be useful for the diagnosis and treatment of COVID-19 [33]. A high neutrophils/lymphocytes ratio implies an aberrant immune response, with increased neutrophils and decreased lymphocytes [34,35]. On the one hand, neutrophil production can be triggered by virus-related inflammatory factors, such as interleukin-6 and interleukin-8, tumor necrosis factor-α, granulocyte colony-stimulating factor and interferon-γ. On the other hand, lymphopenia is common in COVID-19, as mentioned, as a result of direct cytokine-induced inhibition [34]. Some publications even considered this ratio as an independent biomarker for indicating poor clinical outcomes [36], which have significant predictive value in COVID-19 mortality [37].

Regarding the main objectives of this study, a characterization model that easily indicate COVID-19 infection was developed. The implementation of a multivariate statistical bioinformatic instrument can provide valid information about the presence or absence of COVID-19 in this population, using rapid and easily available clinical and blood determinants as variable predictors [38].

The model showed that metastasis was high associated with the presence of COVID-19 in this cohort. Metastasis has been associated with high mortality risk [39] OTEI, but not with the early characterization of patients with COVID-19.

C-reactive protein and age have been previously associated with COVID-19 severity and have been suggested as predictors of COVID-19 progression [40,41]. In addition, a higher number of male subjects presented COVID-19, but female sex was shown to be an important variable for the characterization of COVID-19. These results could indicate that female sex could be an interesting variable for the characterization, but male sex presented high risk of mortality, as shown in the table. Strong evidence of a male bias in COVID-19 disease severity has been hypothesized to be mediated by sex differential immune response against SARS-CoV-2 [42]. The presence of immunosuppression, anemia, COPD and cardiorespiratory disease were shown to be negatively associated with COVID-19-positive patients. These results indicate that subjects with these complications were not positive in COVID-19 in this cohort, probably because these subjects were more cautious to COVID-19 infection due to their complications.

Leukocytes and monocytes levels were shown to be negatively associated with COVID-19 infection. As mentioned above, patients with COVID-19 presented lower levels of leukocytes; therefore, high levels of these cells could indicate absence of COVID-19.

Thus, this cohort showed that using easy-to-obtain variables can rapidly separate patients with a high suspicion of COVID-19, allowing for high medical intervention in order to minimize death possibilities [13]. Moreover, the mortality rates can be also useful for the evaluation and stratification of COVID-19 patients admitted to hospital into different management groups for receiving special medical attention and avoiding COVID-19 fatality as much as possible. In this sense, the mortality caused by COVID-19 could be inferred considering not only hematological determinations, but also inflammatory biomarkers such as C-reactive protein, as shown in the mortality model proposed in the current analysis [43].

Days from onset to admission could also help discriminate patients with COVID-19. However, data referring to the outpatient course of the disease and mortality must be interpreted with caution due to the exceptional nature of the hospital situation during the COVID-19 pandemic in our country.

On the methodological aspect, the study has some limitations. Data collection in two different periods of medical activity could lead to classification biases, given the differences in the hospital context in both periods. The same view could be said regarding the heterogeneity of both periods in temporal terms (12 months vs. 9 months). However, the magnitude of the pandemic and the inability to carry out the studies in a normal context justifies our research, while these factors need further evaluation and validation. In this line, the magnitude of the pandemic and the overload of healthcare personal limited the inclusion of biological variables, such as glucose level, triglycerides, total cholesterol or coagulation parameters, which could complement the model proposed. Additionally, the inclusion of coagulation markers could improve this type of investigations [44]. On the other hand, seasonal viruses were collected over a complete annual cycle to avoid selection bias, while COVID-19 has not yet shown a seasonal affinity, reducing the effect of an heterogenous data recollection [45]. It could be argued that part of the results relies on the differences between hospitals. In this sense, the presence of common protocols and multidisciplinary sessions between the leaders of the strategy against influenza and COVID-19 allowed for a common standard of care in the hospital consortium. On the other hand, the size of the sample, the well-validated techniques to classify the different types of viral infection and the discrimination capacity of the extreme values of the HM COVID-19 scale favor the plausibility of the data [46]. The current results should be considered as proof of concept for the development of future hypotheses. In this context, the difficulty in finding retrospective cohorts of comparison with data on respiratory viruses, especially in the detection of seasonal respiratory viruses other than influenza virus, can make data replication difficult and gives extra value to these results.

As a corollary, the results of this study support the use of easy-to-collect inflammatory clinical variables from patients upon arrival to classify them according to the risk of presenting COVID-19. This finding has applications in different areas, such as hospital management in terms of preventive isolation and patient care as well as for personalized medicine.

## 5. Conclusions

The utilization of easily available clinical and hematological determinations could help to early discrimination of hospitalized patients at high and low risk of presenting with COVID-19, consequently allowing medics to apply the most appropriate medical intervention.

## Figures and Tables

**Table 1:** Clinical and phenotypical characteristics of subjects included in this cohort, categorized according to the PCR results for different respiratory viruses.

Variable	COVID-19 Positive *n* = 313	COVID-19 Negative *n* = 237	Flu Positive *n* = 119	Pan-Viral Positive *n* = 116	Pan-Viral Negative *n* = 166	Global *n* = 951	*p* Value
**Age (y)**	69.5 (14.7)	69.5 (16.3)	66.5 (17.1)	66.9 (17.1)	66.3 (17.1)	68.2 (16.2)	0.256
**Male sex (%)**	198 (63.3)	139 (58.6)	58 (48.7)	51 (44.0)	86 (51.8)	532 (55.9)	**0.001**
**Cardiovascular disease (%)**	33 (10.5)	31 (13.1)	22 (18.5)	26 (22.4)	33 (11.4)	(8.0)	0.593
**Cerebrovascular disease (%)**	18 (5.7)	14 (5.9)	2 (2.5)	3 (2.9)	11 (6.6)	48 (5.1)	0.823
**Cardiorespiratory disease (%)**	44 (14.0)	49 (20.6)	32 (26.8)	73 (62.9)	50 (30.1)	248 (26.1)	**0.027**
**Chronic obstructive pulmonary disease (%)**	18 (5.8)	29 (12.2)	22 (18.5)	26 (22.4)	30 (18.1)	125 (13.1)	**<0.001**
**Diabetes Mellitus (%)**	55 (17.6)	40 (16.9)	13 (10.9)	16 (13.8)	15 (9)	139 (14.6)	0.277
**Chronic hepatic disease (%)**	2 (0.6)	2 (0.8)	0 (0)	1 (0.8)	5 (3.0)	10 (1.1)	0.866
**Dementia (%)**	21 (6.7)	23 (9.7)	1 (0.8)	3 (2.6)	4 (2.4)	52 (5.5)	**0.031**
**Connective tissue disease (%)**	6 (1.9)	5 (2.1)	5 (4.2)	5 (4.3)	15 (9)	36 (3.8)	0.197
**Chronic kidney disease (%)**	10 (3.2)	9 (3.8)	5 (4.2)	4 (3.4)	5 (3)	33 (3.5)	0.967
**Acquired Immuno Deficiency Syndrom (AIDS) (%)**	0 (0)	1 (0.4)	0 (0)	1 (0.8)	0 (0)	2 (0.2)	0.624
**Leukemia (%)**	5 (1.6)	5 (2.1)	2 (1.7)	7 (6.0)	25 (15.1)	44 (4.6)	**<0.001**
**Lymphoma (%)**	11 (3.5)	8 (3.4)	7 (5.9)	4 (3.4)	16 (9.6)	46 (4.8)	0.384
**Solid tumor (%)**	41 (13.1)	19 (8)	13 (10.9)	13 (11.2)	23 (13.9)	109 (11.5)	0.73
**Metastatic tumor (%)**	17 (5.4)	13 (5.5)	11 (9.2)	10 (8.6)	11 (6.6)	62 (6.5)	0.375
**Anemia (%)**	71 (22.7)	54 (23.1)	52 (43.3)	33 (28.4)	16 (9.6)	226 (23.7)	**0.001**
**Hemiplegia (%)**	0 (0)	3 (1.2)	0 (0)	0 (0)	0 (0)	3 (0.3)	0.322
**Onset symptons (days)**	6.4 (6.23)	6.8 (5.4)	4.8 (5.52)	5.8 (6.22)	5.4 (5.25)	6.1 (5.81)	0.095
**Hospitalization (days)**	12.2 (11.9)	9.4 (9.7)	11.3 (9.8)	11.2 (11.9)	14.8 (19.9)	11.7 (13.1)	0.850
**Inmunosupression (%)**	27 (8.6)	19 (8.0)	24 (20.2)	28 (24.1)	69 (41.6)	167 (17.6)	**<0.001**
**Exitus (%)**	71 (22.7)	24 (10.1)	7 (5.9)	4 (3.4)	15 (9)	121 (12.7)	<0.001

*p*-value: *t*-test/Mann–Whitney test or ANOVA/Kruskal–Wallis for continuous variables and chi-square test for categorical variables. *p* value in bold type means significant difference.

**Table 2:** Biochemical and hemogram determinations of patients at the moment of hospital admission.

Variables (at the Moment of Hospitalization)	COVID-19 Positive *n* = 313	COVID-19 Negative *n* = 237	Flu Positive *n* = 119	Pan-Viral Positive *n* = 116	Pan-Viral Negative *n* = 166	Global *n* = 951	*p* Value *
**Hemoglobin (g/dL)**	13.56 (1.99)	13.61 (2.01)	12.55 (2.44)	12.42 (2.33)	11.96 (2.48)	13.03 (2.29)	**<0.001**
**Leukocytes (cells/µL)**	7.703 (4.261)	9.010 (4.847)	10.287 (8.986)	9.873 (6.937)	10.806 (9.260)	9.162 (6.635)	**<0.001**
**Neutrophils, (cells/µL)**	6.199 (4.089)	6.941 (4.318)	7.630 (5.692)	8.046 (6.517)	7.734 (5.640)	7.055 (5.025)	**0.001**
**Lymphocytes (cells/µL)**	961 (497)	1.295 (997)	1.420 (3.103)	1.106 (976)	1.695 (4.333)	1.249 (2.237)	**0.020**
**Monocytes (cells/µL)**	491 (434)	654 (415)	979 (2.845)	696 (696)	1.244 (4.846)	750 (2.312)	0.084
**C-reactive protein (mg/dL)**	120.52 (105.61)	95.38 (99.86)	125.67 (130.91)	110.5 (127.10)	115.22 (120.24)	112.73 (111.41)	0.116
**Neutrophils/Lymphocytes ratio**	8.75 (1.3)	7.88 (0.7)	9.32 (1.6)	9.86 (2.1)	9.11 (2.3)	9.49 (2.6)	**<0.001**

* *p*-value: *t*-test/Mann–Whitney test or ANOVA/Kruskal–Wallis for continuous variables and chi-square test for categorical variables. *p* value in bold type means significant difference

**Table 3:** Logistic regression model with dependent variable PCR test results for COVID-19 and using clinical and inflammatory determinations as important predictors for discriminating between patients with COVID-19 and others respiratory complications.

	OR	95% CI	*p* Value	AUC
**MODEL** ***(R^2^ = 0.13)***				*0.75*
Age > 68 (y)	1.49	(1.08–2.06)	*<0.001*	
Sex female	1.74	(1.28–2.37)	*<0.001*	
Anemia	0.49	(0.37–0.79)	*0.002*	
Immunosuppression	0.31	(0.17–0.54)	*<0.001*	
C-reactive protein > 110 (mg/L)	1.39	(1.00–1.92)	*0.047*	
CODP	0.44	(0.21–0.87)	*0.020*	
Cardiorespiratory disease	0.68	(0.40–1.15)	*0.156*	
Metastasis	2.22	(1.07–4.57)	*0.031*	
Leukocytes > 9000 (cells/µL)	0.54	(0.37–0.76)	*0.001*	
Monocytes > 700 (cells/µL)	0.38	(0.26–0.55)	*<0.001*	

AUC: Area Under the Curve. CI: confidence interval. OR: odds ratio. Collinearity was assessed by variance inflation factor (VIF).

**Table 4:** Comparison of leukocytes levels (neutrophils, lymphocytes and monocytes) by Student’s *t*-test between patients who were positive and negative in COVID-19, showing lower levels of white cells in patients with COVID-19. Patients negative in COVID-19 were considered patients included in groups flu positive and pan-viral negative) Values are expressed as mean ± SD.

Variable (Cells/µL)	COVID-19 Negative (*n* = 472)	COVID-19 Positive (*n* = 313)	*p* Value
**Leukocytes**	9811 ± 7443	7703 ± 4261	**<0.001**
**Neutrophils**	7379 ± 5408	6199 ± 4089	**0.002**
**Monocytes**	855 ± 2766	491 ± 434	**0.016**
**Lymphocytes**	1370 ± 2684	961 ± 497	**0.005**

Values are expressed as mean ± SD. *p* value in bold type means significant difference.

## Data Availability

Not applicable.
